# Supplementary material for: Strong correlations elucidate the electronic structure and phase diagram of LaAlO3/SrTiO3 interface
Source: Nat Commun. 2015 Sep 11;6:8239. doi: 10.1038/ncomms9239 (PMC4647855; doi:10.1038/ncomms9239)
Supplement: Supplementary Information — Supplementary Figures 1-6 and Supplementary References. [file ncomms9239-s1.pdf]

SUPPLEMENTARY FIGURES

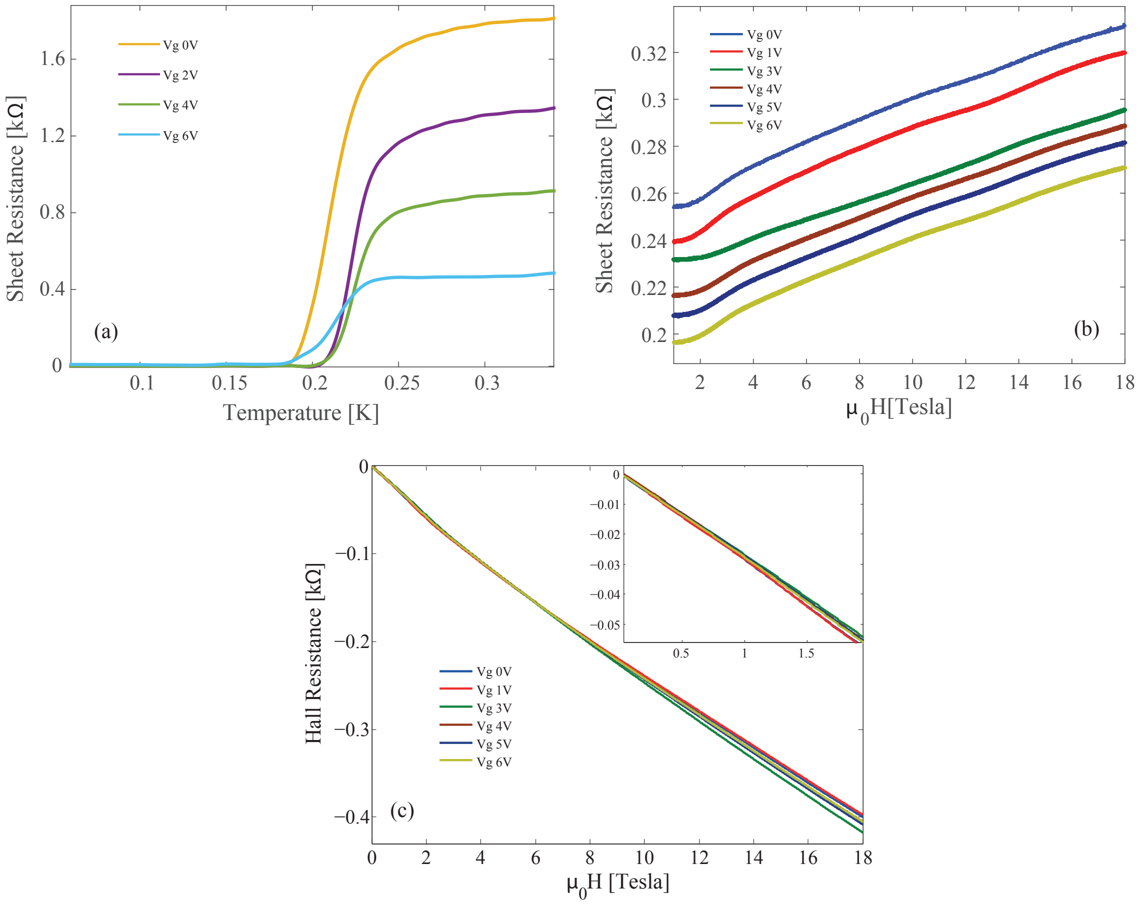

Supplementary Figure 1: Additional measurements of Samples A and B. (a) Sheet resistance versus temperature for various gate biases V_g (Sample A). The transition width is about 20%. (b) Sheet resistance versus magnetic field for various gate biases V_g (Sample B). The gate bias V_g=3V is also shown at the main paper (Figure 2b). All other gate bias plots are shifted for clarity. (c) Antisymetrized Hall resistance versus magnetic field for various gate biases V_g (Sample B). Inset: Zoom-in on the low field regime.

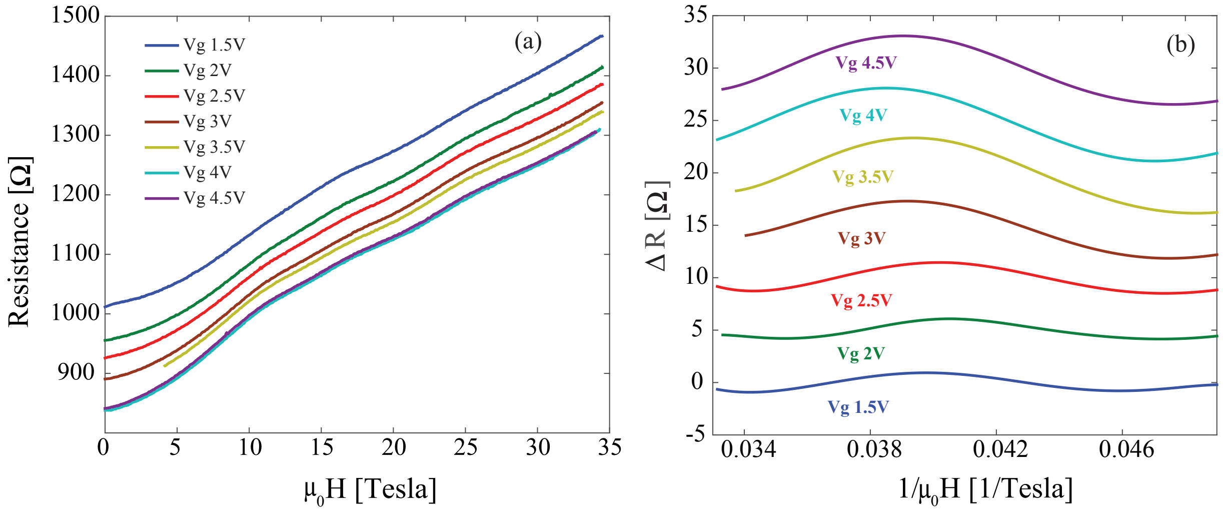

Supplementary Figure 2: Low and High field measurements of Sample C. (a) Resistance versus magnetic field for various fixed values of gate bias V_g. (b) Resistance versus inverse magnetic field after subtraction of a smooth polynomial background for various fixed values of gate bias V_g. Successive curves are shifted by 5Ω for clarity.

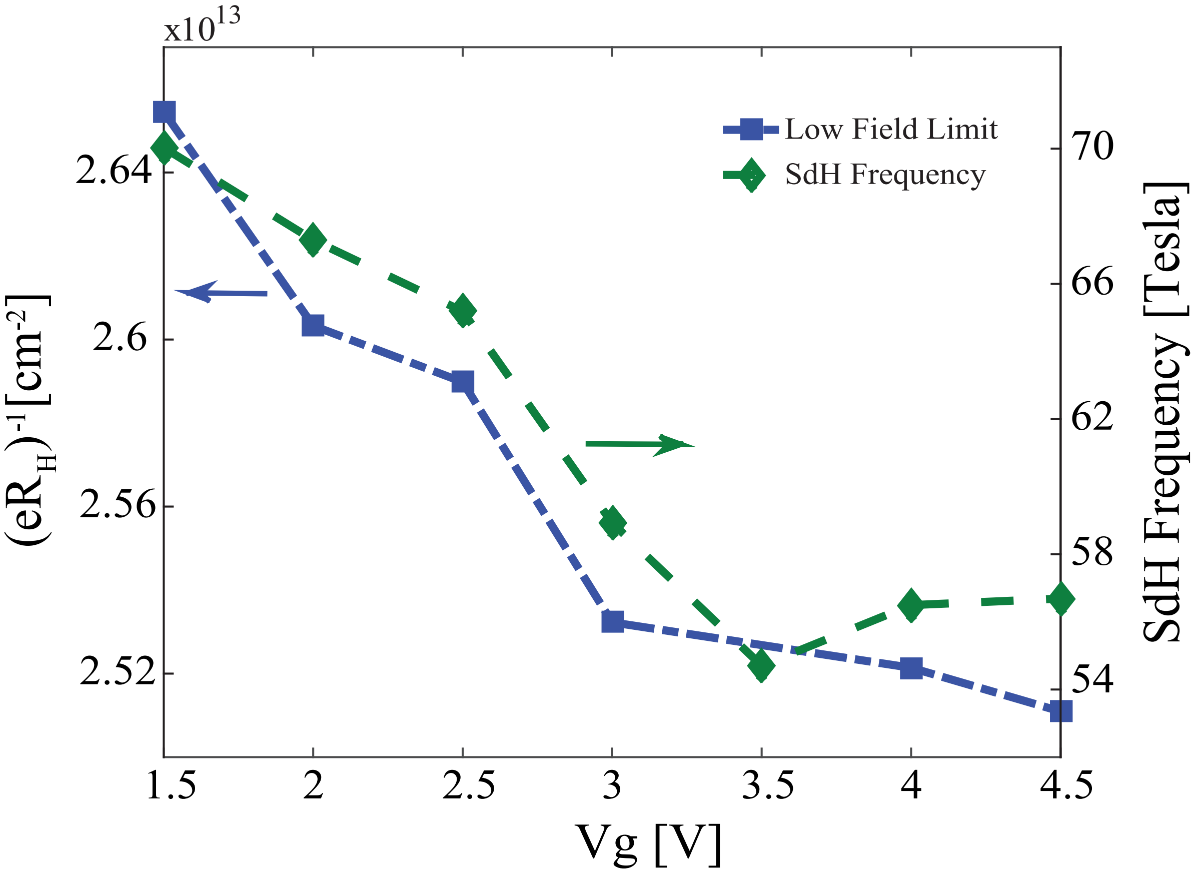

Supplementary Figure 3: A focus on the anomalous regime where the SdH frequency and the inverse Hall coefficient decrease with increasing gate bias for Sample C. Left axis (indicated by a blue arrow): The inverse of the Hall coefficient inferred from a linear fit to the measured Hall data up to 2 T is plotted as a function of the gate bias (blue squares). Right axis (indicated by a green arrow): The SdH frequency is plotted as a function of the gate bias (green diamonds). The SdH frequency is calculated from FFT analysis of the data in Supplementary Figure 2(b). Interestingly, the number of carrier inferred from both measurements decreases with increasing V_g. This reproduces the behavior described in Figure 3(b) in the main paper for the overdoped region with a higher resolution.

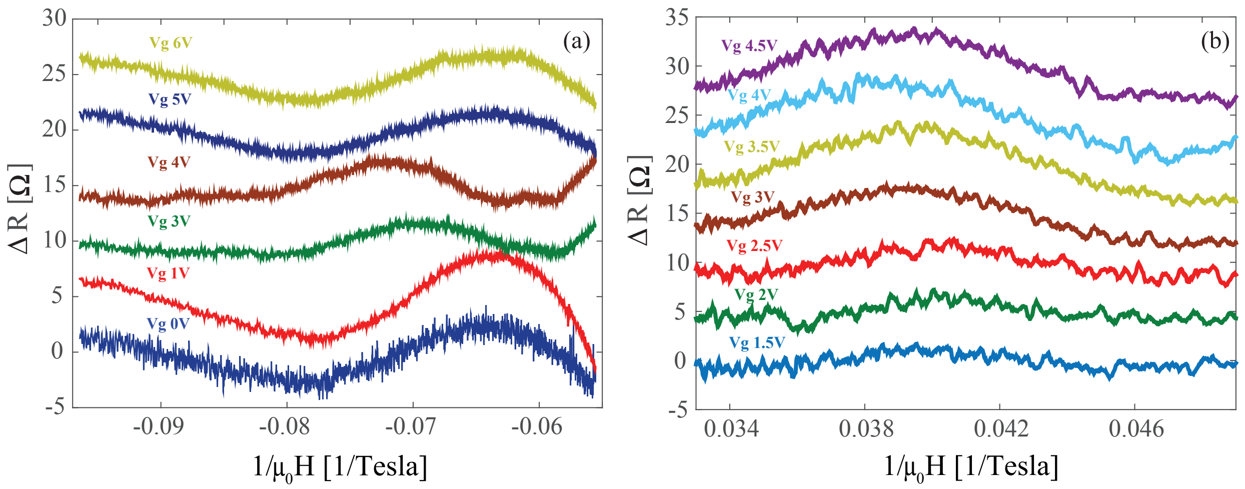

Supplementary Figure 4: Unfiltered SdH data after background subtraction. (a) and (b) Resistance versus inverse magnetic field (Samples B and C) after subtraction of a smooth polynomial background for various fixed values of gate bias V_g. Successive curves are shifted by 5Ω for clarity. These are the unfiltered data which corresponds to Figure 2(b) in the main text (Sample B) and Supplementary Figure 2(b) (Sample C).

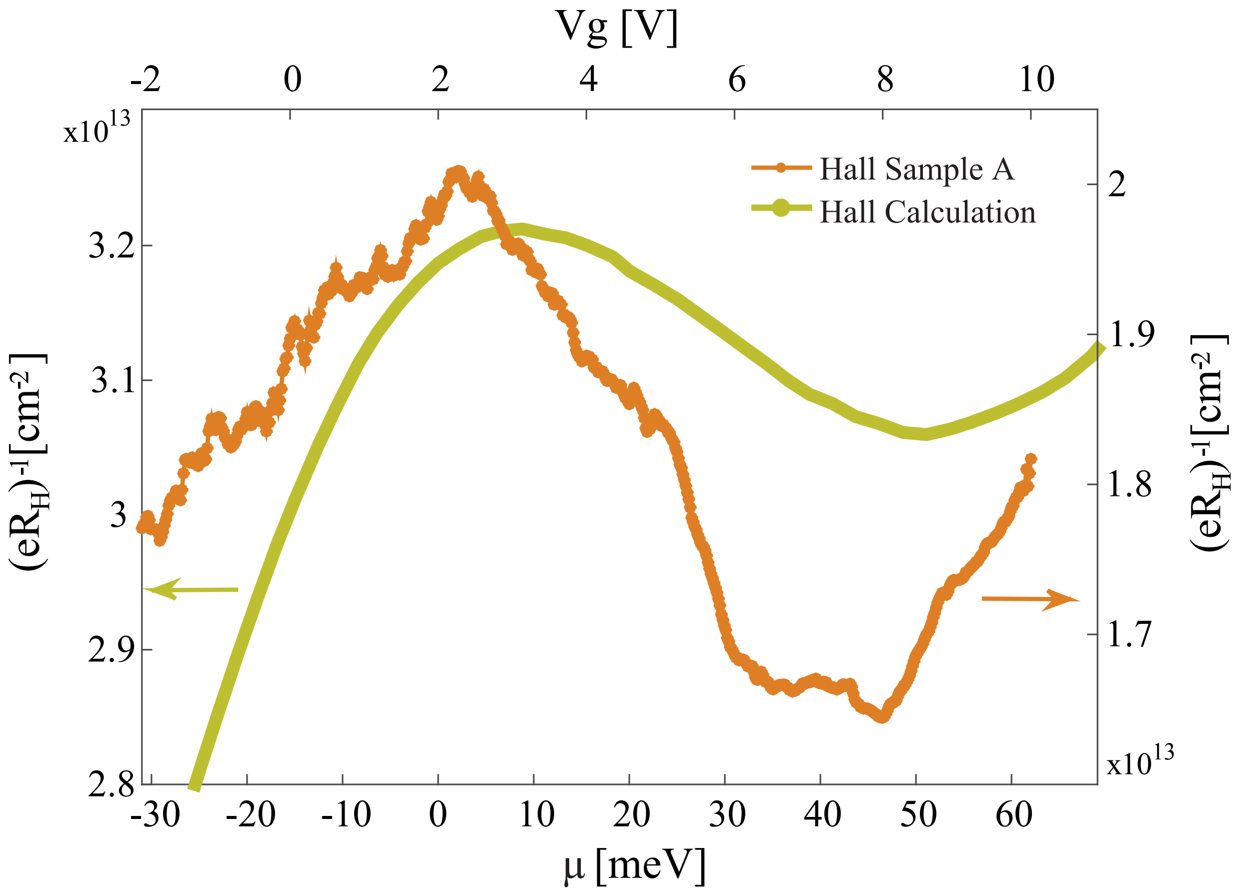

Supplementary Figure 5: Comparing experimental data and theoretical calculations. Bottom-Left axes (indicated by a yellow arrow): Calculated inverse Hall coefficient in the low field limit (described in the main paper) as a function of μ. Top-Right axes (indicated by a brown arrow): The inverse of the Hall coefficient at low magnetic field (Sample A) is plotted as a function of the gate bias V_g. The chemical potential μ was shifted and scaled using the parameters in Figure 5(b) in the main paper to match the location of the maximal T_c between the experimental and calculated data. Our calculation reproduce the nonmonotonic behavior of the inverse Hall coefficient.

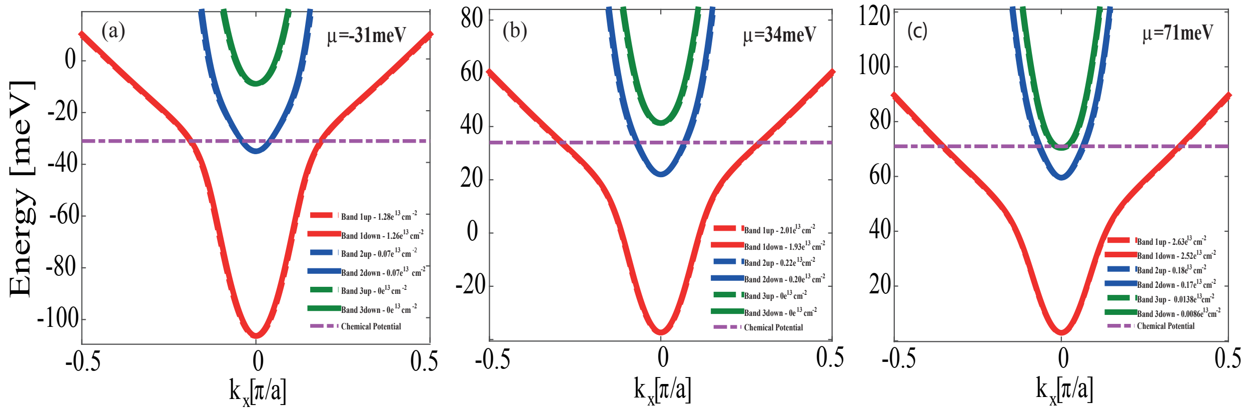

Supplementary Figure 6: Band structure including Rashba spin orbit interaction. (a-c) Calculated band structures using the same parameters as in the main paper and including the Rashba spin-orbit interaction for three different chemical potentials μ.

The Rashba spin-orbit interaction term breaks inversion symmetry, resulting in the following orbital mixing terms :

$$H_R(\mathrm{k}) = \Delta_R \begin{pmatrix} \sin(k_y a)\sigma_x - \sin(k_x a)\sigma_y & 0 & 0 \\ 0 & \frac{t_h}{t_l}\left[\sin(k_y a)\right]\sigma_x - \sin(k_x a)\sigma_y & 0 \\ 0 & 0 & \sin(k_y a)\sigma_x - \frac{t_h}{t_l}\left[\sin(k_x a)\right]\sigma_y \end{pmatrix},$$

where $a = 3.905\text{Å}$ is the $SrTiO_3$ lattice constant, $t_l = 875meV$, $t_h = 40meV$ and $\Delta_R = 5meV$ [1, 2].

The calculated carrier densities for each band are shown. Comparing these results to Figure 4(a-c) in the main paper we can see a very small splitting of the bands which introduces a minute change into the carrier densities. This effect does not change the behavior of the transport properties and the density of states as a function of μ.

SUPPLEMENTARY REFERENCES

[1] Ben Shalom, M., Sachs, M., Rakhmilevitch, D., Palevski, A. & Dagan, Y. Tuning spin-orbit coupling and superconductivity at the $SrTiO_3/LaAlO_3$ interface: A magnetotransport study. *Phys. Rev. Lett.* **104**, 126802 (2010).

[2] Caviglia, A. *et al.* Tunable rashba spin-orbit interaction at oxide interfaces. *Phys. Rev. Lett.* **104**, 126803 (2010).
